# Supplementary material for: Overview of 71 European community-based initiatives against childhood obesity starting between 2005 and 2011: general characteristics and reported effects
Source: BMC Public Health. 2014 Jul 28;14:758. doi: 10.1186/1471-2458-14-758 (PMC4125700; doi:10.1186/1471-2458-14-758)
Supplement: Supplementary file 1 — Additional file 1: List of potentially suitable projects. Description data: Overview of all the 278 potentially suitable projects identified for the survey together with the method with which they were identified, whether they filled in the questionnaire, and if so, whether they met all inclusion criteria. Information from a Cochrane review on preventive programmes targeting childhood obesity (reference no. 11) was added to this overview. (PDF 100 KB) [file 12889_2013_6895_MOESM1_ESM.pdf]

List of potentially suitable projects

Method of identification
1= Suggested by key informants
2= Suggested by other professionals
3= Inventory of previous overviews and existing databases
4= WHO inventory 2008
5= Cochrane review of prevention projects (reference: 8)

Country		Potentially suitable projects	Method of identification	Response	Eligible
**Austria**	**1**	X-team	1		
	**2**	KIG Kinder im Gleichgewicht	1		
	**3**	teen power 10/14	1		
	**4**	Durch Dick und Dünn	1		
	**5**	Rundum gsund	1		
	**6**	Down and up	1		
	**7**	In.Form	1		
**Belgium**	**1**	Fitte School	3,4		
	**2**	VIASANO (EPODE methodology)	3	X	
	**3**	Middle School Intervention	3,5		No (8)
	**4**	Tutti Frutti-project (fruit distribution in schools)	4		
	**5**	My active food triangle	4		
	**6**	Introducting healthy nutrition in special youth care centers	4	X	
	**7**	Healthy behaviour at school Promotion plan	4		
	**8**	Gezondwerken (www.gezondwerken.be)	1		
	**9**	www.hartelijkebuurt.be	1		
	**10**	Zahnhygiene und gesunde Ernährung in Schulen	1	X	
	**11**	Idefics	2		
**Bulgary**	**1**	Health 4 schools	3		
	**2**	Healthy eating in the kindergartens	4		
	**3**	Food and Nutrition Action Plan	1		
**Cyprus**	**1**	Cyprus Healthy Children Program	1		

Country		Potentially suitable projects	Method of identification	Response	Eligible
**Czech Republic**	**1**	"Little Pyramid man"	1	X	No
	**2**	Healthy Teeth	1		
**Denmark**	**1**	Diet in a nutshell (national initiative)	1	X	
	**2**	Shape Up lab	3		
	**3**	The Municipalities Plan against Obesity	3,4	n.a.	Not able to contact
	**4**	Danish: Aktiv rundt i Danmark	3		
	**5**	"Boost - Frugt og grønt"	4		
	**6**	Spaces to move children. Local & Facilities Fund	4		
	**7**	Copenhagen School Child Intervention Study	4	X	
	**8**	6 a day ("6 om dagen")	4		
	**9**	Whole Grain Campaign	4		
	**10**	Get moving	4		
**Estonia**	**1**	Shape Up lab	3	n.a.	Not able to contact
	**2**	Camps for obese children	1		
**Finland**	**1**	Attention to Weight! - Weight Control Program	1		
	**2**	Children's Welfare Project (HYVIS)	1		
	**3**	MUUVIT	2		
**France**	**1**	Together let's prevent childhood obesity"	3		
	**2**	Shape Up lab	3		
	**3**	Program in the Aquitaine region	3	X	No
	**4**	Prevention overweight in preschool children	3		
	**5**	ICAPS	3,5	X (8)	
	**6**	"Health Behaviour in School-Aged Children"	4	n.a.	Not able to contact
	**7**	Plan obésité	1	X	
	**8**	Programme national nutrition santé	1		
	**9**	EPODE	2	X	
		Jouret 2009	5	n.a. (8)	
		Lazaar 2007	5	n.a. (8)	
		Paineau, 2008	5	n.a. (8)	
**Germany**	**1**	Besser essen. Mehr bewegen.	1	X	
	**2**	peb Projekt Junge Eltern - Ludwigsburg	1		
	**3**	FördeKids	1		

Country		Potentially suitable projects	Method of identification	Response	Eligible
	**4**	Lebenslust-Leibeslust	1	X	
	**5**	Aktionsplan Holsteinische Schweiz	1		
	**6**	Projekt T.A.F.F.	1,5	X (8)	
	**7**	CrescNet	1	X	No
	**8**	Kita Vital	1	X	
		Müller, 2001	5	n.a.	No (8)
**Greece**	**1**	The Vyronas study	1		
	**2**	The CHILDREN study	1	X	
	**3**	Paideiatrophi	2	X	
**Hungary**	**1**	HAPPY	1	X	
	**2**	Start with breakfast!	1		
	**3**	Go Healthy! Programme	1	X	
	**4**	Healthy kindergarten in Hungary	1		
	**5**	Eco-School Network	1	X	
**Iceland**	**1**	Everything affects us, especially ourselves!	3	X	
	**2**	6H.is	1	X	
	**3**	Physical activity of Icelandic children	1		
**Ireland**	**1**	Little steps to healthy eating/ living	4		
	**2**	National programmes"Irisch Sports Council"	4		
	**3**	BeActive After-School activity programme	2	X	
	**4**	School Meals Project, Limerick Food Partnership	2		
	**5**	The Gardening Club, Surestart Shantallow	2		
	**6**	Healthy School Food Policy	2		
	**7**	Fresh Fruit in Schools Project	2	X	
	**8**	Pack a Punch	2		
	**9**	Breakfast Club	2		
	**10**	CAWT	2		
	**11**	Cook It	2	X	
	**12**	Action for life	2	X	
		Harrison, 2006 “switch off, get active”	5	n.a. (8)	
**Italy**	**1**	Crescere Felix	1	n.a.	Not able to contact
	**2**	Gaining health	1	X	No
	**3**	Health aging	1		

Country		Potentially suitable projects	Method of identification	Response	Eligible
		Amaro 2006 –Naples (school intervention)	5	n.a. (8)	
**Latvia**	**1**	Project European Healthy Stadia Network	1	X	No
**Lithuania**	**1**	Course of action for School-children Nutrition	1	X	No
**Luxembourg**	**1**	Action plan GIMB	1		
**Malta**	**1**	Girls on the Move	4		
	**2**	Skolasport	4		
	**3**	Summer on the Move	4		
	**4**	Active Youngsters	4		
	**5**	Afterschool sports	4		
	**6**	Sportsbuzz	4		
	**7**	Sportsfun	4		
	**8**	Arti-Sport	4		
	**9**	Sports for all Initiative	4		
**Netherlands**	**1**	Communities in beweging	1		
	**2**	JUMP-in: GGD Amsterdam.	1		
	**3**	Lekker fit Rotterdam:	1,5	(8)	
	**4**	B-fit	1	X	
	**5**	GO-Utrecht	1	X	
	**6**	Familie Lekkerbek	1	X	
	**7**	Gezond Nijmegen	1		
	**8**	Dik en doun in Grunn	1		
	**9**	Voorkomen overgewicht bij kinderen	1		
	**10**	Valthermond Gezond	1		
	**11**	Slagkracht Winterswijk	1	X	
	**12**	B.Slim	1	X	No
	**13**	Gezonde Slagkracht van een Prachtstad	1		
	**14**	Gezonde Slagkracht Woerden	1	X	
	**15**	Lekker in je vel	1	X	
	**16**	De Gezondheidsrace	1	X	
	**17**	Gezond inrichten Gageldonk-West	1		
	**18**	Raalte gezond!	1	X	
	**19**	Wijkgezonder in Zeist	1	X	
	**20**	Sociale activeringscampagne in de RNV	1	X	
	**21**	Gezond in de buurt	1		

Country		Potentially suitable projects	Method of identification	Response	Eligible
	**22**	Samen gezond	1	X	
	**23**	Rivierenland in Balans	1		
	**24**	sCoolsport	1	X	
	**25**	Fit en food	1		
	**26**	On the move - Haarlemmermeer	1	X	
	**27**	JOGG	1		
		Hameling-Baksteen, 2008 - Rhenen-Elst	5	n.a. (8)	
		Singh, 2009 - Do-IT	5	n.a. (8)	
		Veldhuis 2009 – be active, eat right (youth health care)	5	n.a. (8)	
**Norway**	**1**	Physical activity and healthy meals at school programme	4		
**Poland**	**1**	National Programme for Prevention Overweight 2007-2011	1	X	
	**2**	Keep Fit	1	X	
**Portugal**	**1**	Shape Up lab	3		
	**2**	PASSE	4		
**Romania**	**1**	Increase access primary medical prevention services	1	X	
	**2**	I also live a healthy life!	1	X	
**Slovakia**	**1**	Feedback method – with evaluation of body posture	4		
	**2**	Program health at schools in Trebišov district	4		
	**3**	National obesity prevention program	4		
	**4**	School fruit schematic program "Skolske ovocie"	4		
**Slovenia**	**1**	Veter v laseh (Wind in your hair)	4		
	**2**	Razvoj pristopov za spodbujanje zdrave prehrane in gibanja v srednjih šolah	4		
	**3**	FIT Slovenia International	4		
	**4**	Zdrava prehrana, zdrava mladina	4	n.a.	Not able to contact
	**5**	Šole, ki promovirajo zdravo prehrano (NFSI)	4		
	**6**	Pro greens	4		
	**7**	Vzgoja za zdravje (Education for health)	4		
	**8**	Projekt Jabolko (Apple project)	4		
	**9**	Zlati sonček (Golden sun)	4		
**Spain**	**1**	Program Perseo / Programa Perseo	1		
	**2**	Educative Program "5 per day"	1		

Country		Potentially suitable projects	Method of identification	Response	Eligible
	**3**	Program Thao - (Programa Thao- Salud infantil)	1	X	
	**4**	Delta Project	1	X	
	**5**	Children moving (Niños en movimiento)	1		
	**6**	Communitary project in Berriozar	1		
	**7**	DISFRUITA-LA	1		
	**8**	Prevention of obesity in school-age population	1	X	
	**9**	PAIDO	1	X	
	**10**	Program for schools' dining halls in Madrid Community	1	X	
	**11**	Plan Integral de Villanueva de la Cañada	1		
	**12**	Participative project in Extremadura	1	X	
	**13**	NEREU Program (Programa NEREU)	1		
	**14**	MOVI Program	1,5	X (8)	
	**15**	Prevention project obesity	1		
	**16**	PIMSE	1		
	**17**	Move with us: Exercise looks after you	1	X	
	**18**	Program in Molina de Segura	1	X	
	**19**	CAPSA	1		
	**20**	PASEA	1		
	**21**	Integral plan in Murcia	1	X	
	**22**	Córdoba for health	1		
	**23**	Attention in comunity health care centers	1	X	
	**24**	Plan for physical Activity, Sport and Health (PAFES)	1		
	**25**	Strategy PAAS (Estrategia PAAS)	1	X	No
	**26**	Project in Rioja	1		
	**27**	Estrategia NAOS	1	X	No
	**28**	Integral Plan in Andalucia	1		
	**29**	POIBA project Barcelona	1	X	
**Sweden**	**1**	Life in motion	4	X	No
	**2**	Skolmatsakademin (School meal Academy)	4		
	**3**	Runda barn (Överweight Children)	4		
	**4**	Enjoying Life - motion och mat för en friskare framtid	4		
	**5**	SECOPP	4		
	**6**	The Bunkeflo project	4		
	**7**	Familjeviktskolan [Family Weight School]	4	X	No

Country		Potentially suitable projects	Method of identification	Response	Eligible
	**8**	Friska barn – förskolan	4	X	
	**9**	Föräldrastöd för goda mat	4	X	
	**10**	Implementing action plan 2004	4		
	**11**	PRIMROSE	4		
	**12**	SALUT	4	X	
	**13**	Viktiga barn och ungdomar	4		
	**14**	STOPP	3		
	**15**	IDEFICS	3		
	**16**	Pro Greens	3		
	**17**	Gå eller cykla till skolan!	3		
	**18**	Health Equilibrium Initiative	2	X	
	**19**	CBI (not specified)	2		
	**20**	Regional program Vastra Gotaland	2		
	**21**	Childhood obesity program in Jönköpiong County	2	X	
	**22**	SCIP-school	2	X	No
		Marcus, 2009	5	n.a.	No (8)
**Switzerland**	**1**	Ballabeina study	3,5	(8)	
	**2**	Cantonal Intervention Programms	4		
	**3**	Pedibus	4		
	**4**	Aktion Znüni-Box "Gesundes Znüni"	4		
	**5**	prevention project children 0-3 years	4	X	
	**6**	SchoolCatering for Children and Adolescent	4		
	**7**	Kinder-SportstudieKISS	4		
	**8**	Miges Balù	4	X	
**United Kingdom**	**1**	CHOPPS	3,4,5		No (8)
	**2**	Snack right	3		
	**3**	Physical activity to prevent obesity	3,4		
	**4**	Walk to School campaign	4		
	**5**	Bike it & Safe Routes to School	4		
	**6**	Dragon sport	4		
	**7**	RCTadapting US school obesity prevention to England	3		
	**8**	APPLES	3,5	X	No (8)

Country		Potentially suitable projects	Method of identification	Response	Eligible
	**9**	MEND Program (Mind, Exercise, Nutrition… Do It!)	3	X	
	**10**	The Children's Orchard	3	n.a.	Not able to contact
	**11**	Fit 4 Life – Rushmore Healthy Living	3	X	
	**12**	Shape Up Lab	3		
	**13**	Change4Life	4		
	**14**	The East Midlands Declaration – Change4Life"	3		
	**15**	Appetite for life	4	X	No
	**16**	Convenience Stores	3,4		
	**17**	Get Active Northumberland	1		
	**18**	On the Go	1	X	
	**19**	Healthy4Life	1		
	**20**	Healthy weight communities	1		
	**21**	DASH (Do Activity Stay Healthy)	2		
	**22**	What's in Yours?' Healthy lunch boxes	2		
	**23**	Healthy Schools Plus - 'Food Factor'	2		
	**24**	Barnardo's Healthy Schools Play Projects	2	n.a.	Not able to contact
	**25**	Why Weight Matters?	2		
	**26**	Food for Life Partnership (FFLP)	2	X	
	**27**	HENRY	2		
	**28**	Bike It	2		
	**29**	Villa Vitality	2	X	
	**30**	Active Play and Travel: Tackling Obesity	2		
	**31**	Antenatal Obesity Pilot	2		
	**32**	Activ8 Community Gym	2	X	No
	**33**	B Active Scheme	2		
	**34**	Care Pathway for Children under 3	2		
	**35**	Change for Life	2		
	**36**	Child Obesity Clinic: "Mandometer" technology	2	X	No
	**37**	Early Years Family project	2	n.a.	Not able to contact
					Not able to contact
	**38**	Fit for Life	2	n.a.	
	**39**	Health & Well-being	2		
	**40**	Healthy Kids Programme	2		
	**41**	Healthy Weight, Healthy Lives	2		

Country		Potentially suitable projects	Method of identification	Response	Eligible
	**42**	LEAPActive	2		
	**43**	Looking after me - Change4Life	2		
	**44**	Result!	2		
	**45**	Settling In Sessions	2	n.a.	Not able to contact
	**46**	Step Up	2		
	**47**	Tiger Teams	2		
	**48**	Walk to School	2		
	**49**	Schools4Life	2		
	**50**	Tutors models.	2		
	**51**	Fun, Food and Fitness Project	2	X	
	**52**	NCMP team (national childhood measurement programme)	2	X	
	**53**	Bath and North East Somerset	2		
	**54**	Healthy Schools Plus South West	2		
	**55**	Bath and North East Somerset	2		
	**56**	Gloucestershire	2		
	**57**	Poole	2	n.a.	Not able to contact
	**58**	Somerset Healthy Schools Plus programme	2	n.a.	Not able to contact
	**59**	Five/60	2	X	
	**60**	Nottinghamshire county	2		
	**61**	Fit and active families - Catch 22	2		
	**62**	Family Lifestyle club (FLIC)	2	X	
	**63**	GO4it	2		
	**64**	Fun 4 Life	2	X	
	**65**	Make it count	2		
	**66**	Weight Management Centre- lutton	2		
	**67**	Integrated obesity Care Pathway	2	X	
	**68**	NHS Dudley MEND	2	X	
	**69**	Leisure Services	2		
	**70**	School Travel Programme	2		
	**71**	Community Health Champions	2		
	**72**	A live and Kicking	2	X	
	**73**	FRESH	2	n.a.	Not able to contact
	**74**	Fit to Play	2	n.a.	Not able to contact
	**75**	Exercise Referral	2	n.a.	Not able to contact

Country	Potentially suitable projects	Method of identification	Response	Eligible
**76**	Skip4Life	2	n.a.	Not able to contact
**77**	Sport & Play Development	2	n.a.	Not able to contact
**78**	Healthy Schools Programme	2	n.a.	Not able to contact
	Reilly, 2006	5	n.a. (8)	
	Warren, 2003	5	n.a. (8)	No
	Kipping, 2008	5	n.a. (8)	
	Adab, 2008- Beaches	5	n.a. (8)	
	Barlow, 2008 – EMPOWER program	5	n.a. (8)	

n.a. = not applicable
