# Supplementary material for: Overview of 71 European community-based initiatives against childhood obesity starting between 2005 and 2011: general characteristics and reported effects
Source: BMC Public Health. 2014 Jul 28;14:758. doi: 10.1186/1471-2458-14-758 (PMC4125700; doi:10.1186/1471-2458-14-758)
Supplement: Supplementary file 2 — Additional file 2: List of excluded projects and reasons. Description data: Overview of projects that were excluded for analysis and the reasons for exclusion. (PDF 8 KB) [file 12889_2013_6895_MOESM2_ESM.pdf]

List of excluded projects and reasons

Country/project	Reason
Czech Republic / Little pyramid	No process evaluation
France / Aquitaine region	No involvement of target population reported[1] in planning or executing the CBI
Germany / Crescnet	No involvement of target population reported in planning or executing the CBI
Italy / Gaining health	National action plan
Latvia / European healthy	No involvement of target population reported in planning or executing the CBI
Lithuania / Course of action for School-children Nutrition	National action plan
Netherlands / B-slim beweeg	No involvement of target population reported in planning or executing the CBI
Spain / Strategy PAAS (Estrategia PAAS)	National action plan
Spain / Estrategia NAOS	National action plan
Sweden / Scip school	No involvement of target population reported in planning or executing the CBI
Sweden / Family weight	No involvement of target population reported in planning or executing the CBI
Sweden / Life in motion	No involvement of target population reported in planning or executing the CBI
United Kingdom / Active 8 com	No process evaluation
United Kingdom / appetite for live	No process evaluation
United Kingdom / APPLES	Period of implementation before 2005
United Kingdom / ncmp team	No involvement of target population reported in planning or executing the CBI
United Kingdom / Novel treatment	No intersectoral collaboration at the local level

[1]: in the electronic CBI questionnaire
